# Saudi women’s leadership experiences in the healthcare sector: A qualitative study

**DOI:** 10.1371/journal.pone.0285187

**Published:** 2023-09-19

**Authors:** Abbas Al Mutair, Muna Al-Ghuraibi, Yasmine Alabbasi, Fatimah Alguthaib, Alexander Woodman, Alya Elgamri

**Affiliations:** 1 Research Center, Almoosa Specialist Hospital, Al-Hasa, Saudi Arabia; 2 School of Nursing, University of Wollongong, Wollongong, Australia; 3 Department of Medical-Surgical Nursing, College of Nursing, Princess Nourah Bint Abdulrahman University, Riyadh, Saudi Arabia; 4 Department of Nursing, Prince Sultan Military College of Health Sciences, Dahran, Saudi Arabia; 5 Almoosa College of Health Sciences, Al-Ahsa, Saudi Arabia; 6 Department of Social Studies, College of Humanity and Social Science, King Saud University, Riyadh, Saudi Arabia; 7 Department of Maternity and Child Health Nursing, College of Nursing, Princess Nourah Bint Abdulrahman University, Riyadh, Saudi Arabia; 8 School of Health Science, University of Salford, Manchester, United Kingdom; 9 Faculty of Dentistry, University of Khartoum, Khartoum, Sudan; Torrens University Australia, NEPAL

## Abstract

**Background:**

Gender equality in the workforce and the promotion of woman leadership is critical to economic growth and the sustainable development of society and the global community. However, gender diversity in leadership positions is a concern as women continue to be underrepresented. Ensuring equal opportunities in leadership positions in the health sector can help advance the achievement of the sustainable development goals (SDGs).

**Purpose:**

The aim of this study was to explore Saudi women’s perspectives and leadership experiences at senior-level positions in the healthcare sector.

**Methods:**

A descriptive qualitative approach was adopted to address the study aim. This included nine semi-structured interviews with Saudi women who have held leadership positions in the health sector over the past ten years. Reflexive thematic analysis was conducted by adopting the six phases.

**Results:**

The results showed that internal factors, such as qualifications, experience, and the innate qualities of a winner, are the most important factors that contribute to women’s leadership. Women’s role expectations, gender norms, and the patriarchal nature of the community have a negative impact on women’s leadership. One of the new findings of this study was negative attitudes and lack of support from female colleagues.

**Conclusion:**

Women leaders in health care in Saudi Arabia share similarities and differences with women leaders around the world. However, the Saudi community has its own social norms and gender roles that cannot be denied. While Vision 2030 brought a number of positive changes in women’s empowerment that participants spoke of, more research is needed to explore men’s perceptions, which can complete the picture and lead to organizational improvement and changes.

## Introduction

Gender equality in the workforce and the promotion of woman leadership is critical to economic growth and the sustainable development of society and the global community. However, gender diversity in leadership positions is a concern as women continue to be underrepresented [1–4]. Yet globally, women still earn an average of 20% less than men and make up only about 28% of executives across sectors. They face numerous barriers to entry and promotion. Moreover, only about 26% of parliamentarians worldwide are women. In the health sector, women make up about 71% of the global workforce, but only about 26% of leadership positions. According to the Global Health 50/50 study, women from low- and middle-income countries make up 42% of the world’s population but hold only 9% of board seats where global health decisions are made [3]. Moreover, political science shows that when women are represented in legislatures, discussion and implementation of women’s policy priorities also tend to increase [5–7]. Thus, increasing the participation of women as leaders can be seen as a long-term investment in organizational, social, and national prosperity leading to improved health policy, public health, and the quality of life in local and global communities [5–7].

Saudi Arabia is the largest country in the Middle East, with a growing population of 35.84 million, of which 15.14 million are women [8–10]. In 1993, the Saudi Health Care Specialties Commission was established to oversee and regulate medical practitioners’ and institutions’ accreditation. Although women make up more than half of the students and graduates of medical schools, as of 2015, they made up only about one-third of the doctors and nurses on the staff of the Ministry of Health [8–10].

Launched in 2016, Saudi Vision 2030 has provided a strategic framework for transformation through economic diversification and the development of public service sectors such as health, education, infrastructure, leisure, and tourism. One of the central goals of Vision 2030 is the Health Sector Transformation Program, which aims to restructure the health sector to become a comprehensive, efficient and integrated system based on the health of the individual and society [9–11]. As part of Vision 2030, the country has undergone socio-economic changes, including women’s empowerment and inclusion in a number of professions that were previously only occupied by men. In addition, in line with the fifth goal of the United Nations Sustainable Development, Saudi Arabia has secured the right of women to participate in the labor market [11]. Saudi women are expected to play a vital role in this development strategy. As such, the number of Saudi women in leadership positions has increased as the Kingdom pursues new reforms to improve its performance in women’s empowerment and gender equality. The new decrees prohibit gender discrimination in employment [12, 13]. However, unlike Western healthcare workers, Saudi women still make up a smaller percentage of labor force than men in all areas of healthcare except nursing [12]. As of 2018, the medical profession with the highest percentage of female employment in Saudi Arabia was nursing at 61.8%. On average, the percentage of Saudi women employed in the health sector was significantly lower than that of men in all occupations except nursing [8–12].

This study hypothesized that examining the health sector and women’s perspectives could reveal and explain the idiosyncratic barriers women leaders face in Saudi Arabia, including the social and ethical conditions within the country’s cultural boundaries. This study aimed to explore Saudi women’s perspectives and leadership experiences at senior-level positions in the healthcare sector.

## Methods

### Research design

A descriptive qualitative approach was adopted to address the study aim. This included nine semi-structured interviews. Semi-structured, commonly used in qualitative research, allowing for a dialogue between researcher and participant, guided by a flexible interview guide and complemented by clarifying questions, research and commentary. This method allowed for the collection of open data, the exploration of participants’ thoughts, feelings, and beliefs on research aim, reflection on personal and sometimes sensitive issues, presented in results. The interview guide was developed based on the available literature and the evidence on developing guides for qualitative data collection [12–15]. The open-ended questions and probes were prompting enough to keep the discussion active and ongoing, providing a wealth of information about the opinions and feelings of the participants that resulted in a greater variety of responses [14, 15]. The questions explored the attitudes, beliefs and lived experience of participants in the framework of leadership in health care system, barriers and challenges, as well as motivators and stimulus. This research ensured the reliability and validity of a qualitative study through credibility, transferability, dependability, and confirmability [16–18].

### Participants and recruitment

This study applied the purposive sampling technique, that is, identify and select participants who are particularly knowledgeable or experienced and ready to convey experience and opinions related to the study aim [17]. The sample size was determined by approaching the saturation point. Thus, these were Saudi women:

- Holding senior positions in the public, teaching or private healthcare sector in Saudi Arabia for the past ten years or having her own business in health care sector
- Intermediate of fluent knowledge of English to avoid translation bias and misinterpretation of what was meant and what was said or translated. This allowed to avoid possible biased interpretation by the research team, when analyzing data. Participants’ thoughts and ideas were recorded and typed as they were expressed. Whereas, when translating from Arabic, an inevitable bias might arise, which would weaken the credibility and transparency of the study.
- Having at least bachelor’s degree.

Potential interviewees were contacted by phone or email, clearly presenting and describing the purpose of the study, including data collection and storage procedures. It was explained to potential interviewees that data would be collected in accordance with good clinical practice and that no personal information or data would be disclosed. Participants were explained that they had the right to withdraw at any time and were asked to sign the informed consent. As a result, n = 9 participants agreed to take part in this study.

### Data collection

Data was collected using semi-structured interviews to capture the unique experiences of the participants. Prior to the interview, interviewees were informed in writing about the study objectives, methodology, and data protection. Researchers also explained the risks, benefits, and expected outcomes of the research project. After which participants granted their written informed consent. Participants were given the choice between either face-to-face or teleconference interviews. The interview guide was prepared based on the literature review, questions and props were ordered chronologically from the general to the specific. The interviews were conducted by the principal investigators and the co-investigators. In total, 9 interviews were conducted before reaching the saturation point. Out of the 9 interviews, 4 were conducted face-to-face and 5 by teleconference. Before the interview, the respondents were reminded about the study’s objectives, methodology, and data protection. The researchers also explained the risks, benefits, and expected outcomes. Data collection started once the participants gave their written informed consent. Participants were given a choice between face-to-face or teleconference interviews. The principal investigators and the co-investigators conducted the interviews. It was originally intended that the time for each interview would be extended as prolonged engagement is critical, allowing respondents to spend more time explaining their living experiences in Saudi culture, the everyday world, to better understand behaviors, values and explain their thoughts. As a result, the interviews lasted from 60 to 150 minutes, an average of 75 minutes.

The interviews were conducted by the principal investigator and co-investigator who had the experience of conducting mixed-methods studies. The interview process was a balance between flexibility and ensuring that the conversation did not stray too far from the aim of the research. Much of the interview was devoted to exploring the views and thoughts of the participants, asking them to share and compare experiences, and to discuss the extent to which they agree or disagree with the proposed question. The timing and pace of the interview were taken into account so that by the end all questions were covered in sufficient detail. The interviews were audio-recorded, and notes were taken on a portable computer.

### Reliability and validity

Credibility was built through long-term engagement to obtain a full and deep understanding of the participants’ culture, language, and perspectives. The findings were sent to all participants for comments and possible remarks [15–18]. Their comments were included in the final reports. Transferability was ensured by a detailed description of the data collection and procedures, allowing future researchers to reproduce this data in their environment. Dependability was addressed by good documentation of the data analysis process. The researcher kept the raw data, i.e., the audio recording, with field notes, for the possible audit procedures. Confirmability was established when the collected data were checked and rechecked throughout data collection and analysis to ensure results would likely be repeatable by others [15–18].

### Ethical considerations

Ethics approval for this study was obtained from the Almoosa Specialized Hospital Institutional Review Board (IRB) Log Number (ARC-21.05.06) and Princess Nourah Bint Abdulrahman University IRB Log Number (21–0259). Written informed consent was obtained from participants who voluntarily participated in the study interviews. All participants received a copy of their signed consent, and the researcher kept the original. The research was conducted following the Declaration of Helsinki, amended in 2013.

### Data analysis

Reflexive thematic analysis was conducted by adopting the six phases outlined by Braun and Clarke (2006) [18]. This approach facilitated the analysis and helped to identify and focus on important aspects of the thematic analysis, moving back and forth through the phases as needed. Hence, from the outset, the researcher recognized that the six-phase process is a set of recommendations, not rules, to be applied flexibly according to the study’s data and aim [17, 18].

1. This phase started with initial engagement with the data, i.e., transcribing the audio-recording of interviews and reading. Becoming familiar with the collected data through audio recordings, transcripts, and notes taken during data collection was the first and vital step in the analysis. Familiarization allowed the research team to immerse in the collected data and ensure that the full depth and breadth of the data set were considered equally. Recurring phrases, thoughts, and ideas related to the study aim were highlighted. Notes were made on the margins of transcripts.
2. The coding phase began, and an inductive approach to data-driven coding was used. Coding was performed using MAXQDA software version 2020. Mostly semantic coding was performed. However, several emerging codes were identified at this phase. Because codes are fundamental to themes, the team worked systematically on the entire dataset, giving equal attention to each data element. Care was taken to keep the codes concise but detailed enough so that they could be self-contained and inform the underlying commonality between themes and the aim of the study.
3. Once all the data was coded, the research team began looking for themes. In doing this, the focus was on considering and analyzing how codes can be combined according to common meanings to form themes and sub-themes. This included combining multiple codes that share a similar underlying concept or data characteristic into one code. At the end of this phase, a thematic map, i.e., a mind map, was produced by comparing codes and data items relative to themes and sub-themes.
4. The fourth phase of the analysis, a review of potential themes, required a recursive review of themes in relation to the encoded data elements and the entire data set. The themes and sub-themes have been revised according to the coded data extracted from the transcripts. This was done by re-reading all the passages on each theme and checking them for consistency. As a result, a revised thematic map was produced that captured the most important elements of the data in relation to the aim of the study.
5. Phase five of the analysis aimed to define and name themes and sub-themes. The research team reviewed the analysis to give a further reflection and deeper interpretation of the analysis. Subsequently, the themes were fully developed. All themes were brought together to create a lucid narrative consistent with the dataset’s content and informative in relation to the study aim. As a result, the final themes and sub-themes.
6. The sixth phase was to complete and report what was found, a process that requires a recursive approach to writing a report. At this phase, the changes documented in all phases were combined and re-verified by the research team and presented as the study results.

## Results

Socio-demographic analysis of study showed that of nine participants five were married and had children, two were widowed with children, one was married and had no children and one was single. As shown in Table 1, most of the participants were specialized in general medicine (n = 6).

**Table 1:** Characteristics of study sample (n = 9).

**Marital status**	5 Married with kids
	2 Widowed with kids
	1 Married with no kids
	1 Single
**Educational background**	6 Medicine
	1 Biochemistry
	1 Dentistry
	1 Business administration
**Specialty**	1 Family Medicine
	1 Obstetrics and gynecology
	1 Neonatology
	1 Internal medicine
	1 Infectious disease
	1 Project management
	1 Hospital care administration
	3 Quality and patient safety

Semi-structured interviews were conducted with nine participants and six themes derived after data analysis with their sub-themes to be presented as part of results: (1) enablers of a woman leader, (2) obstacles in the way of a leader, (3) impact of the community and the conservatism, (4) being a woman in the workplace, (5) being a successful leader, (6) heading toward the golden era of Saudi women.

### 1.1 Enablers of a woman leader

This theme captured the factors that Saudi women felt were enablers that helped and prepared them to be healthcare leaders. This theme included three sub-themes: (i) innate winning qualities, (ii) qualifications, (iii) philosophical/religious approach to life, (iv) social support, (v) workplace support.

#### 1.1.1 Innate winning qualities

This sub-theme reflected the innate qualities Saudi women had at an early age and during the mid-career path that prepared them for leadership positions. Participants reported being distinguished and top students in the school and when started their medical education.

“*So*, *always in school*, *I was really always number one and looking to be*.. *on the top of the class and then when I did go to medical school*, *I also graduated top of my class*.*”*

(Participant 1: 10)

At the same time, they often reflected and admitted their failures or loss. They saw mistakes and loss as lessons that allowed to benefit and find more creative solutions. Participants revealed that their path has not been smooth or full of success, but they have had inner qualities that helped them learn from those mistakes and that have helped them continue their careers.

“*I got through a lot of situations*, *lot may see it as failure or losing or etc*. *For me*, *I see them as lessons*…*Thanks to God for every lesson*, *every failure*, *every trip*… *one can benefit from all of them and can come out with more creative solutions*.*”*

(Participant 3: 86)

Participants further emphasized the importance of having special personal qualities and character that did not allow them to stop at obstacles to success, such as being passionate, hardworking, and persevering. Thus, they considered these traits the cornerstones and, the most important victorious inner qualities that helped them to be leaders.

“*I know my personality*, *my character*, *I don’t stop in front of any obstacle*, *I don’t stop*, *I don’t think about it*, *I just overcome it and go*.*”*

(Participant 4: 26)

#### 1.1.2 Qualifications

This sub-theme reflects the importance of having good qualifications, sufficient knowledge, and enough experience to achieve the leadership position. Participants made it very clear that they would never become leaders in the health sector unless they combined these three characteristics, with professionalism in academia and training being paramount.

“*So*, *talking about the leadership enablers*, *the first thing is the professionalism which is academic and training*.*”*

(Participant 2, 66)

Participants also recognized the role of continuous education and self-development, especially in the administrative field, to keep pace with peers and colleagues. Thus, despite their leadership position, they continued to improve their knowledge through certifications and courses.

*None of us have been born as administrator*, *we are leader yes*, *but we are not administrative worker*, *so you have to improve that administrative part in you*, *you have to study you have to read you have to join courses workshop*.

(Participant 4, 82)

#### 1.1.3 Philosophical/religious approach to life

This sub-theme reflected the particular approach to life shared by the participants. They had a philosophical and sometimes religious approach to life. They believed that what you sow is what you reap, and a person’s duty is to give, not take. They further noted that while there may be many plans, God corrects and amends the path. There are also environmental factors that influence this leadership path.

“*It’s what’s going around*. *It’s coming again*. *It’s a really the best thing in your life is to give*, *not to take*.*”*

(Participant 5: 7–8)

“*And I got to know now that you really make plans*, *however your plans are modified by the path that God created for you and by environmental factors*.*”*

(Participant 6: 13)

#### 1.1.4 Social support

This sub-theme covered the social support women leaders received from their families. All participants recognized that social support is critical to being a leader. In addition, participants acknowledged that of all the support they received, the support of their father and their husband was most valued and considered paramount.

“*The full support I received was from my parent*. *They were my everything*, *all the support*, *all the backups*.*”*

(Participant 4: 12)

“*And honestly*, *this is to my father and to my husband*. *And without both of them*, *and of course my mother “god bless her soul”*. *My father*, *when I got married*, *the only condition was just let Mageda continue her studies*. *And my*, *my husband*, *honestly*, *he was really behind this*. *He was really very supportive*.*”*

(Participant 5, 29)

#### 1.1.5 Workplace support

This sub-theme captured the support for a woman leader at her workplace. According to the participants, the enablers include having supportive male managers who trust them and give them the chance to learn and prove themselves. One participant explained that by saying:

“*The head department*.. *was like a father for us he really supported me*.. *the kind of support*: *you’re doing great don’t worry*. *If you face a problem*, *we will solve it just send me what you need I’m with you and he never interfere or ask what I’m doing*, *it was really amazing*.*” (Participant 4*: *44)*

The next enabler in the workplace was reported to be the positive climate of the workplace. Different institutes and hospitals were mentioned by the participants as having a positive climate that give them the chance to lead. When asked to elaborate on what they mean by positive climate as workplace they mentioned how the organization and workflow is organized, structured, supportive and nurtured. Participants further underlined by the way a woman leader is treated by her male colleagues in terms of respect and attitude.

“*It’s structured*, *it’s organized*, *you get developed*, *and you get nurtured*. *I think you get developed as a young graduate*, *you need to be developed*, *you need training in the environment that is supportive*, *you need support*, *they give you the basic skills I think*, *ok*. *That what was available there*.*”*

(Participant 6: 15–16)

“*I am the only female*, *amazing amazing*, *I’m receiving the full respect*, *they are considering my opinion about the things*, *I am writing and discussing*, *I am the first one always at the meeting find my name the first one attending*, *fully supported and everybody is happy about this*.*”*

(Participant 4, 101)

### 1.2 Obstacles in the way of a leader

This theme covered the challenges Saudi women face to take on leadership positions in the health sector. These obstacles were classified into internal obstacles’ sub-theme.

Although not as a negative connotation, most of the participants perceived motherhood as an obstacle. Participants believed that the problem was not motherhood but the lack of a family-friendly system, which often forced them to choose between starting a family or professional success. They felt the system was designed to create a seeming conflict between these two responsibilities. One participant explained that she had to quit her job at one stage because of the system.

The next obstacle was that being a woman was considered a big challenge in leadership, which many participants highlighted. Hence, another personal obstacle was the lack of self-confidence or self-awareness that prevented them from pushing to a leadership position. As a result, they tend to wait their turn, while men often take a position without waiting to be asked.

“*I had to resign from my job because of my third pregnancy*. *I got through difficult circumstances at that time since I had a cesarean… high risk cesarean section premature baby*. *They didn’t agree to give me vacation other than the birth vacation which is 42 days*, *and my daughter was premature*.*”*

(Participant 2: 14)

“*But in practice*, *the biggest challenge I found is being a woman*.*”*

(Participant 7., 35)

“*Women are not really into leadership position maybe because they don’t ask like men*, *we don’t we tend not to ask we tend to wait until the position comes to us*, *so women do not ask as for positions or for being the lead leading role as much as the our male counterpart*.*”*

(Participant 1, 16)

### 1.3 Impact of the community and the conservatism

This sub-theme covered the impact of culture and society on women’s leadership in Saudi Arabia prior to women’s empowerment by laws and regulations. Most participants emphasized the stereotype of a woman’s role in the community and social expectations that a woman would marry and stay at home. The participants mentioned that the main problem of the community is the attitude towards women who can be superior to men. In fact, another obstacle was mentioned as the physical appearance of men and women. Thus, if a person is short and thin, their leadership abilities may be questioned or underestimated.

“*From my experience*, *I can find it sometimes difficult for men in our community and culture to get orders form female*, *sometimes*.*”*

(Participant 8: 62)

“*It’s a very important point*, *if you are short and thin is different than if you are tall and huge regardless of being female or male*. *Physical appearance also impacts so you have to be aware of all the perceptions*, *you have to be able to deal with all the perceptions*, *perception of appearance and perception of being male and female… and many other stuffs other than that*.*”*

(Participant 6: 135–137)

Being a conservative community, in general, was considered as a critical factor in shaping the community’s perspective not only about working women in general but specifically about women working in healthcare and as a leader.

“*In my family*…*and some of people of my family think that me as a lady*, *shouldn’t work*… *and I’m from a conservative family*… . *So*, *me being in a work which requires me being in the work for late hours or sometimes traveling*…*transferring and etc*., *sometimes it’s not acceptable to be*.*”*

(Participant 3: 46)

“*In the Saudi society*, *and people kept coming to my mother and asking her*, *are you serious*? *Are you going to let her study medicine*, *she is going to be working with men*, *she’s going to have night shifts*, *how come she is going to sleep outside the house*? *That was the culture that time*.*”*

(Participant 9: 15)

Essentially, this point of view is inherited from the women themselves. One participant shared her experience of running for the highest leadership position in her organization. She lost the election to a male colleague, but found that not even Saudi women voted for her.

“*… to my surprise Saudi female did not vote for me*, *and when I asked one of my best friends like you did not vote for me you know*, *and she said yeah because you do not have the experience men are more experienced men*, *they know better men*.”

(Participant 1: 16)

### 1.4 Being a woman in the workplace

This theme captures issues related to gender in the workplace, especially those that affect the achievement of senior management positions in the health sector. This theme has two sub-themes: ‘gender-related issues’ and ‘communication and socialization.’

#### 1.4.1 Gender-related issues

This sub-theme covered participants’ experiences and perceptions of gender issues such as inequality and the glass ceiling phenomenon, an unrecognized barrier to career advancement, especially affecting women and minorities. All participants agreed that their path as a woman aspiring to leadership has been challenging. They believed the workplace was male-dominated.

At the same time, some felt that jobs in Saudi Arabia were free of gender bias, and government support promoted the inclusion of women in general.

“*As a matter of fact*, *we are privileged in Saudi Arabia that there is no difference in the salary pay between man and women as in US*! *Thanks to the recent government’s vision that support women and created more job opportunities to them*.*”*

(Participant 7: 43)

#### 1.4.2 Communication and socialization

This sub-theme covered the aspect of communication and socialization of women’s leadership. Participants agreed that mutual respect is a solid foundation for communication or socialization. However, participants differed in their opinions when asked about the difference between interacting with a female and a male colleague. Some of them thought it was harder and more sensitive to deal with women, while others thought it was harder to deal with a male colleague because he might underestimate her and show some resistance. However, one participant felt these judgments cannot and should not be generalized because the issue is situational.

“*Dealing with respectful people who respect your mind*, *was critical for me*, *yeah*. *I mean*, *I don’t like to work with qualified people*, *who do not respect you or respect your existent*, *not only as a woman*, *but also as human being*.*”*

(Participant 5, 156)

“*I consider them as a situational*, *interactional positions*. *Female to female might be a challenge*, *male to female might be a challenge*. *Each in its own way*… *Situational*… *Can I say this one is more than the other one*? *No*, *each one has its unique situation*. *Female to female*, *yes*, *there might be jealousy*. *Yes it can happen*, *female to male*, *may be doubting your expertise*, *not convinced that you can be my manager*. *So it differs*, *there is challenge in both situations*. *So I look at it as a challenge and challenge*… *How you can deal with this challenge and how you can deal with the other challenge*.*”*

(Participant 6: 168–169)

Some of them felt that the problem was not that they were women, but environment, which often made it difficult to communicate. When asked to describe the reaction of their male colleagues to a female leader, some participants said that the men were unhappy about it. In fact, some men even refused to accept being led by a woman.

“*He was heard by someone saying*: *“I don’t accept that a woman is my boss”*, *but he was a subordinate*, *so he was reporting to me whether he likes it or not*!*”*

(Participant 7.: 37)

“*Some of them*, *they didn’t feel comfortable*, *so they politely asked to be transferred from the administration and I accepted this*… *in the opposite*, *I encouraged them*… *I said if you find a better opportunity*, *you can move*.*”*

(Participant 3: 50)

One participant raised an interesting point about communication and socialization. She highlighted that women tend to socialize less than their male counterparts, negatively impacting them, as social gathering is a cornerstone in networking, finding mentors, and sponsorships.

“*Women tend not to socialize with her workers which is actually opposite to what people think*. *A woman is more conservative in socializing with their superior or with their coworkers*, *but men is different*, *men they tend to ask*, *they tend to go out places*, *they play golf together they play tennis*.. *they tend to make more communication with their superior more than woman*, *there is a barrier that she is my boss*, *I am not going to go*, *but men*, *no*, *they break these barriers and they communicate better in this way*.*”*

(Participant 1: 32)

### 1.5 Being a successful leader

This theme reflected the participants’ leadership style and how they defined the issue of being a female leader in a senior management position. For many participants, leadership was not related to positions and the chair; it had a deeper meaning. When asked about leadership qualities, they suggested that while a person may be a natural leader with innate leadership abilities, they must work on themselves to be convincing leaders. In addition, they all agreed that being a leader is hard work, harder than it looks, often accompanied with stress and challenges.

“*The chair will not make you a leader*, *the leadership will not be by chair*. *Too many were seated in the chair and left and no one knew about them*.*”*

(Participant 9: 97)

“*I think leadership is both*, *inherent and acquired*. *So some genetic factors play a role but a major part of leadership is acquired through life and work experiences in addition to professional achievements*. *Self-confidence comes from excellence in profession and good communication with people that helps in inspiring others and making you a leader*.*”*

(Participant 7, 24)

“*So*, *the feeling was intense stress at the time of being a director*… *multitasking was challenging*! *Leading 6 departments and following up on their progress and quality projects*, *in addition to solving many of their interpersonal conflicts kept me busy*. *Some subordinates were difficult to satisfy while others were supportive*.*”*

(Participant 7, 39)

Regarding leadership style, participants reported different approaches and stated there is no standardized, right, or wrong leadership style. In addition, participants noted that whatever leadership style is chosen, culture and values must be respected, although this is challenging. One common point expressed by the participants regarding women’s and men’s leadership styles was that most believed that the difference is not related to gender but to personality.

“*Leadership in my opinion is to inspire people so they like to follow you*. *Excellence in your own specialty makes a good reputation about you*, *but also assisting others to excel*, *guiding & communicating frequently with them leave them inspired & taken care of*.*”*

(Participant 7: 68)

“*Work from within the culture*. *You enforced your values but from within the culture*. *You know Just putting*. *Okay*, *you want to go there*, *and you are here*. *We don’t just decide to jump there*. *You work consistently towards the goal*, *working consistently from within the culture*, *I think is important*.*”*

(Participant 6: 133)

“*I don’t think there is really major differences I would say maybe personality differences so I could be different than you as leader but we are both females*, *so I don’t think it’s a there is something in gender that make a female leader is different than a male leader at the same position*.*” (Participant 1*, *18)*

However, some participants felt there was a general difference between women and men in leadership styles, where women had better leadership qualities and were generally more supportive of the organizational culture. Furthermore, some participants believed that women have a great leadership style because they are hardworking, more democratic, persistent, creative, tactful, and dedicated.

“*I think women think of empowering others more*,.. *so I think we have the tendency to empower the others more than men in a role of leadership and responsibilities …*. *We empathize more*, *we can understand the situation and empathize with others more than men*.*”*

(Participant 8: 96–97)

“*I see female leadership and I myself*, *they always have the way to do things*, *I do not remember a female leader said I don’t know or cannot manage things*. *Most of quality management in the hospitals are females*.*”*

(Participant 5: 200)

“*There is a difference*, *of course*. *Most men assume a directive or authoritative leadership style*, *they may listen to your opinion but at the end*, *the final decision is their*! *Most men are directive in general*, *yes*.*”*

(Participant 7: 49)

On the other hand, one participant suggested that although leadership success does not depend on gender, men are determined in leadership and can achieve better results.

“*To be honest*, *the man*, *if he’s good he’s good*, *whenever they have vision for something they can do it for you*, *OK you see improvement you see quality of work*,.. *you see an improvement in the hospital as man if he’s good*.*”*

(Participant 4, 80)

### 1.6 Heading toward the golden era of Saudi women

This theme captures the transformation in women’s empowerment under Vision 2030 from the participants’ perspective. This theme resulted in three sub-themes: (i) living the transformation, (ii) legislation and women’s empowerment (iii) future leaders.

#### 1.6.1 Living the transformation

This sub-theme looked into the real transformations that participants have experienced in terms of women’s empowerment and how they compare their time to the current period in Saudi Arabia, and the impact of these changes. Participants agreed that there had been a huge change in Saudi Arabia regarding women’s empowerment. The first major moment in the history of women’s empowerment and leadership was when King Abdullah promoted the education and training of women in various fields. This was considered a defining moment for women’s rights, after which Saudi women entered the labor market. However, a groundbreaking and ever-changing moment has arrived with Vision 2030. The nature of women’s empowerment has been closely linked to the community’s response to these dramatic changes, which is slowly becoming more tolerant towards women leaders.

“*Thanks God*, *we have gone through transformation now and we have better opportunities*. *There is a real empowerment to women*, *we see a dramatic change in decisions*, *in policies in the Kingdome of Saudi Arabia*.*”*

(Participant 3: 20)

“*I think the era the new Millennium you could really say from 2005 when King Abdallah was in power there was a moment empowerment started back then women empowerment is started*, *I would say in 2005–2004 when more options started opening for women like engineering school the more international world*, *they work in in ministry of foreign affair that they start working on other ministries*.*”*

(Participant 1: 22)

“*When Prince Mohamed bin Salman and King Salman they issued the roles and the vision and all the changes that have happened*.*”*

(Participant 9: 29)

“*I think the transformation and the vision of 2030 have impacted the culture in a positive way*, *so ummm looking into women empowerment in 2004 and now*, *there is a big difference*. *I mean people now will accept women in multiple leadership roles*, *even in some*, *mean we use to have some departments and areas where only men are working*, *now more acceptable to work in those departments*. *So*, *I think introducing women to the work environment and setting targets to empower women and has played a major role*.*”*

(Participant 8: 153–154)

In addition, many participants suggested that the community is becoming very flexible and easily adapts to changes. When asked to compare the current situation with the old days, they were sure that these days are much brighter than the old days of women’s rights and gender equality than their days. Participants believed that much bigger changes were coming to get women active and in leadership positions.

“*I think the community was adjusted automatically to these changes*. *So*, *I think if you compared to the Saudi community five years ago from now*, *you will see huge change and I think it’s becoming faster*.*”*

(Participant 3: 30)

“*I think their time is the true time of equality*. *we can see now the woman in Saudi Arabi*, *they are now holding high positions*, *even positions that we have never heard of before*, *… every day we see name with qualifications and young ladies really empowered*.*”*

(Participant 9: 76)

“*My daughter and her friends now they go to attend conferences outside the kingdom*. *There is a big difference*.*”*

(Participant 9: 80)

“*Because as we said*, *there were a lot of obstacles previously and very minimal enablers*. *Now*, *in the last four five years*, *it’s started to happen*. *If you wait*, *you will see*, *you will have many more*. *You know what I mean*? *It is just a matter of time because we have broken the barriers*, *the barrier is broken*.*”*

(Participant 6: 253)

#### 1.6.2 Legislation and women’s empowerment

This sub-theme reflected participants’ views on official steps to empower women in Saudi Arabia in terms of legislation and regulations. Participants suggested that Vision 2030 paved the way for Saudi women. Women’s empowerment legislation has been very well received. Essentially, participants living in the era before women’s empowerment were amazed at the power and boldness of these laws.

“*I think*.. *the political has paved the way for us*, *and they put us in position*, *and they open a different positions different field basically they did pave the way*.”

(Participant 1: 24)

“*I mean you’re looking at it now just like amazing what happened in the last 20 years in terms of development in this field so back then there weren’t really any policies at the institution level*.*”*

(Participant 1: 12)

One of the most prominent examples that had a profound impact and was frequently cited by participants was driving. In addition, laws relating to domestic violence, child abuse, and workplace harassment were considered very strong and courageous laws that empowered women and protected them at home and beyond.

“*Driving*, *for example*, *now women can drive and ability to reach any area they want to reach*.*”*

(Participant 8: 160)

“*Of course*, *I think if you look into legislations’ that support women in general for instance in 2013 the first legislation and protection from violence and abuse came by and that legislation was one of the strongest legislations because it criminalizes the domestic violence and that was really it’s not just a good move it’s a great leap in the field of women empowerment and protection of women*.*”*

(Participant 1: 26)

#### 1.6.3 Future leaders

This sub-theme covered the advice that current leaders give to girls who are on their way to leadership. Participants stated that while women’s empowerment comes with great opportunities, it also comes with great responsibilities that should be taken seriously. They also suggested that Saudi Arabia’s unique expertise in women’s empowerment, top-down approach, investments, and bold legislation have all brought attention to Saudi women nationally and internationally. While the path for the next generation has been paved, it will not be free from obstacles. Thus, future leaders must be mentally prepared to overcome difficulties and focus their attention on overcoming challenges, and not see them as barriers that stop the path.

“*It is our role now is to walk through that path and to build as we are walking and I think the future lies in the hands of women*, *so women has really to continue working hard um and the security they have in their hands*, *the trust that was given by our political leaders Mohammed bn Suliman*, *it has to be like taken seriously*.*”*

(Participant 1: 24)

“*I think the Saudi woman were under the spotlight*, *under the spotlight because people think we do not have our rights*, *we are not really helping in the development or country*, *now we are still under the light but in a different light*, *the light is that we are really uh helping in in in development of the country*, *it’s really 50/50 is working with men and improving and getting to the vision of 2030 so I think women are on the right track so their role is really to work that track and build it*.*”*

(Participant 1: 44)

“*Don’t think of barriers as obstacles that you cannot deal with*. *Continue to be focused on the goal*. *Don’t put barriers as obstacles and stop*, *continue to be focus on the goal and the more important advice is make use of what is being provided for you*. *In the last three*, *four years*. *This is a dream*… *what is being provided is a dream*. *So they should use it*.*”*

(Participant 6: 259)

Participants believed that empowering one girl could change the world. However, they also believed in the importance of balancing family and work. Future leaders should not have to choose between family and career, while challenges should be seen as new opportunities to get up and move forward. When asked where they see Saudi women in the next ten years, an attempt to reach the moon was mentioned.

“*Empowering one girl can change the world*, *so that’s why I say never underestimate the worth of yourself*. *One person can change the world*, *this is how change happen in the societies*. *This is what happening*. *In every house there is a girl empowered that make you empower the society*.”

(Participant 9: 112)

Nothing impassible, you just break it down and you would find your way.

(Participant 5: 198)

“*If you become something in the community*, *you can do it and you can be a wife and you can live your life normally*, *so it’s very important that this conviction doesn’t exist which I have touched from a lot of the young generation which is today if I want to become this then I need to give up on that so it’s either this or that*… *I mean like I have to choose*.*”*

(Participant 3: 99)

“*Believe in yourself because if you don’t know one will that you can do it and you will do it*, *even if you faced challenges every challenge you learn from*. *And how people say that you shape your experiences*, *you shape it by the bad experiences*.*”*

(Participant 2: 126)

“*We might get to the moon*!*”*

(Participant 8 Participant 8: 198–200)

## Discussion

Globally, women leaders face social and cultural challenges that often define and hinder their career potential in various professions, including healthcare [1–3]. However, less is known about the factors and determinants associated with the growth and development of women leaders. This was one of the first and unique contributions that explored Saudi women’s perspectives and their under-representation at the top-level of healthcare leadership through semi-structured interviews.

The results of this study indicated that women leaders believe that some of the most important factors contributing to women’s leadership are profession and personal qualifications, innate winning qualities, family and workplace support. These findings are consistent with earlier studies by Abalkhail (2017) and Alexander and Lopez (2018). The authors concluded that self-awareness, self-esteem, strength, and weakness are among the main constructs of true leadership in line with family support [19, 20]. In addition, a positive workplace climate is critical to the success of a female leader. Therefore, addressing structural issues and norms in the workplace at the organizational level is necessary to lead to a positive climate. Moreover, moving from individual expectations to organizational-level strategies and system-level change is an imperative way to support women [21, 22]. This evidence from the literature was supported by participants in the current study, who felt that one of the main factors was the climate in the workplace, including relationships with colleagues, male or female. Thus, the participants further clarified that when males neglect them, this is one problem, but the ignorance or fear expressed by female peers was a bigger issue as they often lacked the simple support of a female colleague. This finding confirms previous research that women’s underrepresentation in leadership positions is not due to women’s lack of desire to grow, but rather, compared to men, women are less optimistic due to interpersonal factors and less support from colleagues [23].

According to earlier research, women in medicine face the same challenges in advancing to leadership positions as other colleagues [21–23]. This study found that some women face prejudices that equate leadership with masculinity, preventing them from taking on higher positions. This finding is especially pronounced in the Saudi community, where traditionally, roles in Saudi culture are patriarchal [22, 23]. As shown in this study, some female leaders in this study were often hesitant to make decisive decisions and often tended to consult with their husbands and/or father, especially since most consider their husbands and/or fathers to be leaders in their industries. Thus, despite an increase in the number of women enrolling in all levels of education and in leadership positions, evidence suggests that women in leadership positions experience personal barriers that hinder their effectiveness as leaders. Therefore, in the future, it will be important to conduct regional studies to identify employment difficulties and barriers to career advancement for women [21–23].

It can be assumed that the factors contributing to women’s empowerment in this study can also be considered challenges requiring further study. In particular, more research is needed to explore the perspectives of men, family members, or colleagues, how they see the changes taking place in Saudi Arabia, and the role of women in these changes. Based on this holistic approach and needs assessment, educational and learning activities within the organization and higher education can be developed. This will allow male and female leaders to work together rather than compete and to respect thoughts expressed based on their added value, not gender [21–24].

Vision 2030 aims to comprehensively transform the health sector and restructure it into an efficient and integrated system based on value-adding care, ensuring transparency and improving health services [9–11]. Moreover, the empowerment of Saudi women is at the heart of the Vision 2030 reform program; to increase women’s participation in the labor market from 22% to 30%. As shown in this study, since Vision 2030 introduction in 2016, Saudi women leaders have witnessed rapid and inclusive growth and development that has reshaped the economic, social, and cultural landscape, making Saudi society more tolerant and receptive [9–11]. However, given the qualitative nature of this study and the thoughts expressed about men’s perspectives, it is recommended that additional quantitative studies be conducted to examine how the number of women leaders in the health system has increased and whether the changes are qualitative or quantitative [25].

In addition to the main aim of this study, it has led to several new findings that require further study, preferably with the inclusion of women leaders from different generations. The factors, challenges, and types of support mentioned by the participants in this study were, in most cases, associated with previous studies, including those that women sometimes do not ask for or negotiate for senior leadership positions [26]. However, the reasons for this similarity or contrast can be quite different depending on the culture of the country and the environment in which women grew up. Therefore, in addition to the opportunities created by Vision 2030, this initiative can serve as a focus for researchers to explore whether the rapid changes taking place following the law and legislation affect the character, thoughts, and traditional values of different generations and their perception of women leaders in the health care sector [28, 29].

Long-standing male dominance in this area forces men to act as gatekeepers, and no woman can rise to the top without being promoted or supported by a man. These findings of long-standing male dominance are consistent with previous data from Saudi Arabia and international data [22, 27–30].

Leadership and leadership styles were detailed by participants who valued leadership approaches over positions, consistent with previous research [14, 22]. Thus, the participants in this study described how their leadership styles and approaches helped them earn respect and recognition in their organizations. In addition, it was clear that their leadership style was determined by the inevitable interaction between their personalities, work environment, and culture [22]. In terms of gender, as in previous studies, conflicting thoughts were expressed: some participants in this study believed that women could be better leaders than their male counterparts in some situations, while others considered women leaders to be less capable than their male counterparts because of their leadership style [21–23].

Finally, the future of women’s leadership in Saudi Arabia as part of Vision 2030 has proven to be decisive and multifaceted. Due to the great transformation in recent years, women’s leadership has become much more dominated by supportive factors, consistent with previous studies among women in Saudi Arabia [31]. Thus, the obstacles mentioned in this study can be considered as factors requiring further research. This will require multi-stakeholder participation and cooperation, which can lead to changes in the attitudes and behavior of women, men, and society [31, 32].

It can be argued that gender norms are changing in Saudi Arabia in line with Vision 2030 that has implemented women’s empowerment on a solid basis. Moreover, these changes are not limited to the labor market; there is a positive shift in culture and society regarding the leadership of Saudi women. However, regulatory actions will be more useful if they studied from men’s perspective. This should be further supported by a sustainable cultural change to advance gender equality in the organization and society in line with Vision 2030. Such an integrated approach will create a strong supportive culture, allowing women to expand their experience and develop their leadership potential.

### Limitations of the study

This was one of the first qualitative studies of Saudi women’s perspectives on leadership in the health sector; hence, it had inevitable limitations. The main limitation was data triangulation, that is, the use of various data sources in the study, including time, space, and people. Therefore, mixed-method studies with more participants are recommended for future research to reduce the shortcomings and errors that occur when using a single method. In addition, future research may consider using focus group discussions to express diverse thoughts, make arguments, and explore the aim of the research from different perspectives of participants from different backgrounds.

## Conclusion

Despite the rapid changes and transformations in Saudi Arabia as part of Vision 2030, the unique culture and community of Saudi Arabia impact women’s leadership. In addition, the power of social norms and gender roles cannot be denied. However, since its implementation, Vision 2030 has brought a number of positive changes that could be permanent and promising opportunities for women’s empowerment and could provide a solid foundation for women’s leadership in Saudi Arabia. Based on this study’s findings, several policy instruments can help advance women in leadership positions, including training and promotion initiatives in healthcare organizations and medical and nursing schools. Networking is critical as it will allow women in leadership positions to share their experiences and provide advice to young leaders to help them achieve their leadership goals.
