# Correction: Saudi women’s leadership experiences in the healthcare sector: A qualitative study

**DOI:** 10.1371/journal.pone.0316872

**Published:** 2024-12-30

**Authors:** Abbas Al Mutair, Muna Al-Ghuraibi, Yasmine Alabbasi, Fatemah Alghadheeb, Alexander Woodman, Alya Elgamri

The fourth author’s name is spelled incorrectly. The correct name is: Fatemah Alghadheeb. The correct citation is: Al Mutair A, Al-Ghuraibi M, Alabbasi Y, Alghadheeb F, Woodman A, Elgamri A (2023) Saudi women’s leadership experiences in the healthcare sector: A qualitative study. PLOS ONE 18(9): e0285187. https://doi.org/10.1371/journal.pone.0285187.

The affiliation for the fourth author is incorrect. The correct affiliation is not indicated. Fatemah Alghadheeb is not affiliated with #1 but with: King Saud University Medical City, Riyadh, Saudi Arabia.
